# Two-Plate Splintless Repositioning in Bimaxillary Surgery: Accuracy and Influence of Segmental Osteotomies in a Consecutive Single-Centre Cohort

**DOI:** 10.3390/jpm15120588

**Published:** 2025-12-02

**Authors:** Hylke van der Wel, Tom Lucas Zwijnenberg, Johan Jansma, Rutger Hendrik Schepers, Haye Hendrik Glas

**Affiliations:** 1 Clinic for Virtual Surgical Planning, 3D VSP B.V., Hoogwatum 2, 9906 TD Groningen, The Netherlands; h.vd.wel@3dvsp.com (H.v.d.W.); h.h.glas@3dvsp.com (H.H.G.); 2 Department of Oral and Maxillofacial Surgery, Expertcenter for Orthofacial Surgery, Martini Hospital Groningen, Van Swietenplein 1, 9728 NT Groningen, The Netherlands; jansmaj@mzh.nl (J.J.); r.h.schepers@mzh.nl (R.H.S.)

**Keywords:** orthognathic surgery, bone plates, patient specific osteosynthesis, computer aided design, minimally invasive surgery

## Abstract

**Background/Objectives**: The primary objective of this study was to evaluate the accuracy of maxillary repositioning using a two-plate patient-specific osteosynthesis system. The secondary objective was to determine whether accuracy is influenced by the number of maxillary segments. **Methods**: A retrospective single-centre cohort study was conducted on patients undergoing bimaxillary orthognathic surgery with a maxilla-first two-plate PSO system. Virtual Surgical Planning was performed based on the Cone-Beam Computed Tomography (CBCT) data of the patient, with patient-specific plates being designed and manufactured accordingly. Postoperative CBCT scans (7–10 days post-op) were registered to the preoperative plan, and deviations in translation and rotation between the plan and results were determined. Sub-group analyses were performed on one-, two- and three-segment maxillary osteotomy patient groups. **Results**: The inclusion criteria were met by 61 patients, of whom 47 were included for analysis (mean age 27.9 ± 9.4 years). Sub-millimetre median translational accuracies were found: anteroposterior 0.7 mm, transverse 0.4 mm, vertical 0.6 mm. The median rotational deviations were ≤1° for yaw and roll, and 1.6° for pitch. Accuracy was consistent across the one-, two-, and three-segment osteotomy groups. **Conclusions**: The two-plate PSO system is clinically accurate in bimaxillary surgery. There is no significant difference in accuracy between one-piece and segmental osteotomies of the maxilla when using the two-plate system.

## 1. Introduction

Bimaxillary orthognathic surgery is a fundamental procedure used to correct dentofacial malalignments. A successful outcome is critically dependent on the predictable and accurate execution of the pre-operative plan. ‘Accurate’ is often defined as a final result with a deviation of less than 2 millimetres in any direction, as greater discrepancies compromise the final result and are considered clinically significant [1,2]. Additionally, ‘midline accuracy’ is defined as perceivably asymmetric when the deviation is more than 1 mm [3]. rotational accuracy, 2 degrees [4,5] and 4 degrees [6,7] are used as clinical acceptability thresholds.

The advent of three-dimensional Virtual Surgical Planning (VSP) has fundamentally transformed the field of orthognathic surgery, enabling the digital simulation of osteotomies and jaw repositioning to within millimetre(s) deviations. Patient-Specific Osteosynthesis (PSO) serves as the most accurate link for translating this digital blueprint to the patient [6]. While the four-plate PSO system is the most widely studied and utilised method for maxillary fixation, increased attention has been directed towards the concept of Minimally Invasive Orthognathic Surgery [8,9,10], leading to a recent re-evaluation of the necessity for four-point maxilla PSO plate fixation [11,12].

Established evidence provides a foundation for the concept of using two plates at the nasomaxillary buttress. Initial clinical studies supporting this idea evaluated the use of traditional bendable osteosynthesis plates for two-plate anterior fixation [13,14,15,16]. Murray et al. were among the first to directly compare the stability of Le Fort I osteotomies fixed with two bendable plates at the piriform aperture versus the four traditional plates, finding no significant difference in postoperative skeletal stability between the two fixation groups, with observed relapses averaging less than 1 mm [13]. Later, Mavili et al. demonstrated the mechanical adequacy of two-plate fixation by confirming that two plates at the nasomaxillary buttress provide sufficient stability to prevent anteroposterior movement of the maxilla [14]. This was reinforced by Susarla et al., who provided evidence of stable outcomes over a one-year follow-up period after using only two-point nasomaxillary fixation for maxillary advancement [15]. Beyler et al. further supported these findings in a study that confirmed using two plates bilaterally at the piriform aperture results in postoperative stability and is comparable to placing four plates, with no significant differences in vertical or sagittal relapses [16].

Building upon this concept, two anteriorly placed patient-specific osteosynthesis plates may offer a less invasive alternative to 4 PSO plates [11,12]. Alfaro et al. introduced a novel two-plate PSO system designed to facilitate a minimally invasive approach, reporting potential advantages such as reduced operation time and decreased surgical morbidity with adequate accuracy [11]. The clinical feasibility of the two-plate PSO system was further supported by van der Wel et al., who indicated comparable accuracy to the conventional four-plate patient-specific method [12].

Despite the documented findings, a knowledge gap persists. The existing literature is limited by small sample sizes and has not yet explored procedures like segmental osteotomies. This study seeks to bridge the gap by presenting a comprehensive, single-centre evaluation of the two-plate PSO system. In doing so, it aims to extend current understanding of the use of patient-specific two-plate systems in orthognathic surgery. The primary outcome is the accuracy of maxillary repositioning. Additionally, the study investigates whether repositioning accuracy following orthognathic surgery is affected by the number of maxillary segments.

## 2. Materials and Methods

### 2.1. Study Design and Setting

This retrospective cohort study was conducted at the Department of Oral and Maxillofacial Surgery of the Martini Hospital Groningen (The Netherlands). The hospital adopted the two-plate Patient-Specific Osteosynthesis (PSO) system for orthognathic surgery in September 2024. Patients who underwent bimaxillary orthognathic surgery using the two-plate PSO system between September 2024 and September 2025 were retrospectively identified by reviewing surgical records. The criteria for including or excluding patients are presented in Table 1. The study was approved by the Ethics Committee of the Martini Hospital Groningen (approval number: 2025-006, dated 3 March 2025).

### 2.2. Data Acquisition

As part of the routine clinical work-up, pre-operative data was acquired, including Cone-Beam Computed Tomography (CBCT) scans, intraoral dental scans and 2D photographs of the patient in a natural head position. These datasets facilitated the basis for virtual surgical planning (VSP), performed by an experienced technical physician (H.v.d.W. or H.H.G.) using the Materialise software (Materialise Enlight, version 5.0; Materialise NV, Leuven, Belgium).

### 2.3. Virtual Surgical Planning

**Figure 1:**
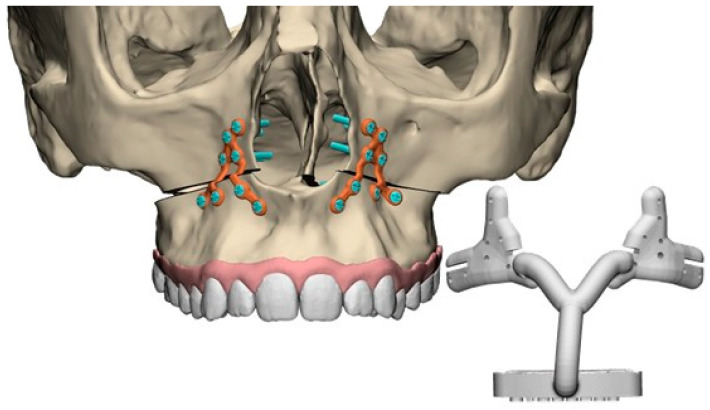
Digital design of the inverted Y-shaped Patient-Specific Osteosynthesis plate and the corresponding surgical drilling guide. The design shows the plate positioned at the nasomaxillary buttress, providing two-plate fixation of the maxilla.

In consultation with the operating surgeon, the VSP was approved and used to design the two-plate PSO system (including a guide). Screw positions were determined to ensure placement in regions with adequate bone thickness, avoiding dental roots, maintaining a safe distance from the planned osteotomies and allowing minimal invasive access. The PSO plates had an inverted Y-shape, with eight screws per plate (See Figure 1). Four screws were placed superior to the osteotomy plane and lateral to the piriform aperture. The other four screws were placed inferior to the osteotomy plane, with two in the dorsal extension and two in the ventral extension of the inverted Y. Regarding the segmental osteotomies, if the osteotomy cut passed between the arms of the plate, the two arms of the plate were connected across the osteotomy gap to bridge the maxilla segments. The osteosynthesis plates were 3D-printed using Ti-6Al-4V Grade 23. Surgical guides were designed to facilitate accurate positioning of the two-plate PSO system, as shown in Figure 1. These guides incorporated a combination of tooth-borne and bone-borne supports for stability during surgery. The position of the osteotomy plane was visible in the guide. The use of two anterior fixation plates and the corresponding guide system enabled both guided drilling and plate placement through a smaller vestibular incision, supporting a minimally invasive approach. The PSO system, including guides, was manufactured by 3D VSP (3D VSP B.V., Bierum, The Netherlands).

### 2.4. Surgical Procedure

**Figure 2:**
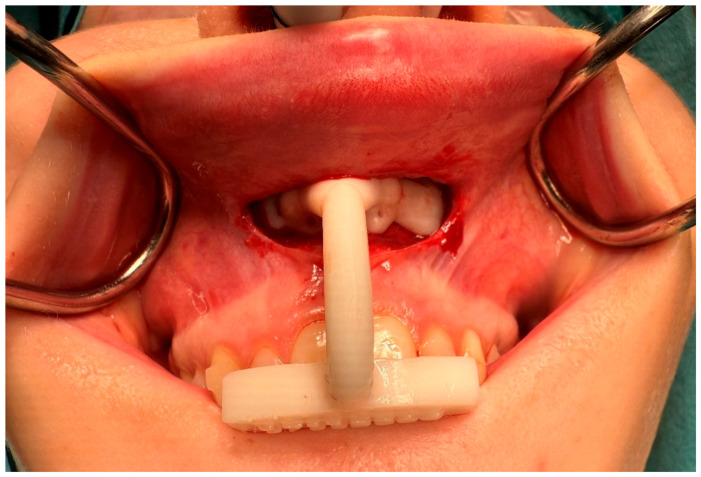
Intraoperative image showing the tooth-bone-borne drilling guide, inserted in its unique orientation with a limited upper vestibular incision. The guide is used to drill the screw holes and perform the osteotomies at their virtually planned positions.

All the surgical procedures followed the maxilla-first protocol. A limited upper vestibular incision was used to access the maxilla (see Figure 2). An intermediate splint was available for each patient as a backup in case conversion to conventional splint-based surgery was required. The maxilla was fixated using the patient-specific plates and 1.5 mm MaxDrive screws (KLS Martin, Tuttlingen, Germany). Following maxillary fixation, mandibular positioning was guided by a final occlusion splint. All surgeries were performed by a maxillofacial surgeon experienced with PSO in orthognathic surgery (J.J. or R.H.S.).

### 2.5. Post-Operative Evaluation

**Figure 3:**
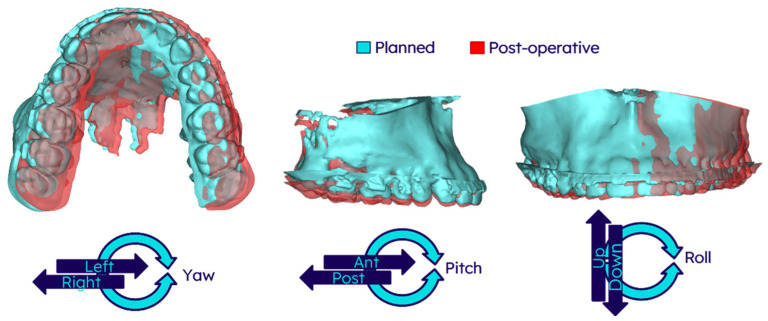
Three-dimensional (3D) representation of the deviation analysis of the maxilla. The planned position (blue mesh) and the post-operative position (red mesh) of the maxilla are superimposed to illustrate deviations. The lower panel defines the translational and rotational movement directions relative to the natural head position coordinate system established during the Virtual Surgical Planning. Abbreviations: Ant, anterior; Post, posterior.

Post-operative CBCT scans were acquired 7 to 10 days after surgery. The position of the maxilla was evaluated using a systematic image registration protocol in Materialise Mimics (Materialise Mimics, version 27.0). First, voxel-based registration was used to align the post-operative skull to the pre-operative dataset, ensuring consistent head positioning. A second voxel-based registration was then performed to align the pre-operative maxilla to the post-operative maxilla to isolate its movement relative to the skull. To quantify positional accuracy, the differences in landmark positions were measured in millimetres (mm) as translational (x, y, z) and degrees (°), as well as rotational (pitch, roll, yaw) movements (See Figure 3). The translations and rotations were calculated within the natural head position coordinate system, as determined during the VSP. To account for intra-arch variation in accuracy in segmental Le Fort I osteotomies, five cephalometric landmarks on the maxilla were used: one located between both central incisors, two on the canines, and two on the upper first molars. The mean positions of the canines and the first molars were used for analysis purposes. These measurements allowed for a detailed assessment of the surgical outcome relative to the planned maxillary position.

### 2.6. Statistical Analysis

Statistical analyses were performed using IBM SPSS Statistics, version 30 (IBM Corp., Armonk, NY, USA). The normality of the data was assessed using the Shapiro–Wilk test. Since the data for all three groups were not normally distributed, the Kruskal–Wallis test was used to determine significant differences. The Spearman correlation test was performed to check for any significant correlation between planned translations/rotations and achieved outcomes. A threshold of *p* < 0.05 was considered statistically significant. Each patient’s outcome was categorised as optimal, good, or suboptimal based on predefined accuracy thresholds (Table 2). While no clear consensus exists on acceptable limits, several studies suggest that deviations under 2 mm and 2° represent good surgical accuracy [1,2], whereas others propose a rotational threshold of up to 4° [6,7].

## 3. Results

### 3.1. Study Data

Out of the 61 patients who met the inclusion criteria, 47 were included in the final analysis after excluding 14 patients due to movement artefacts in the CBCT, which made accurate analysis impossible. The mean age at surgery was 27.9 ± 9.4 years, see Table 3. Among the included patients, 31 underwent a one-piece, 10 a two-segment, and 6 a three-segment maxillary osteotomy. Table 4 shows the planned movement in all directions. The median planned anteroposterior movement was the largest, with 5.0 mm anteriorly. The median planned rotation was 0.1° counterclockwise (CCW) for the roll, 0.9° CCW for the pitch and 0.3° CCW for the yaw.

### 3.2. Accuracy of the Surgery

The absolute deviations from the planned position at the upper incisor are shown in Table 5. Postoperative analysis revealed submillimetre median translational deviations: 0.7 mm anteroposterior, 0.4 mm transverse, and 0.6 mm vertical. Rotational deviation was 0.8° for the roll, 1.6° for the pitch and 0.5° for the yaw. Deviations are quantified at the upper incisor landmark and presented as median values with interquartile range (IQR).

All the median deviations, except for the pitch, remained below one millimetre or one degree. To assess pitch rotation further, the patients were divided into two groups based on the direction of the planned rotation—counterclockwise (CCW) and clockwise (CW). The median planned pitch for the CCW cases was 3.04°, with a realised median rotation of 1.80°, resulting in a median absolute error of 1.67°. Regarding the CW cases, the median planned pitch was 3.80° and the realised median 2.42°, with a median absolute error of 0.94°.

A Spearman’s correlation analysis was performed on each direction to test the correlation between the magnitude of planned movement and the realised error (see Table 6). A moderate positive correlation was found for the roll (*r* = 0.311, *p* = 0.034) and pitch (*r* = 0.316, *p* = 0.031).

### 3.3. Accuracy of Segment Osteotomies

No significant differences in post-operative deviations from planning were found between the one-piece and segmental Le Fort I osteotomies at any landmarks or in any direction. Table 7 shows the absolute deviations at three landmarks: the upper incisors, upper canines and the upper first molars.

Palatal expansion was calculated for the 16 segmental cases from the distance between the upper first molars. The accuracy in achieving the desired palatal expansion is shown in Table 8. The realised palatal expansion (2.7 mm, interquartile range (IQR) 1.3–3.2 mm) was less than the planned palatal expansion (3.0 mm, IQR 1.8–3.8 mm). The absolute median error between the realised and planned expansion was 1.2 mm.

**Figure 4:**
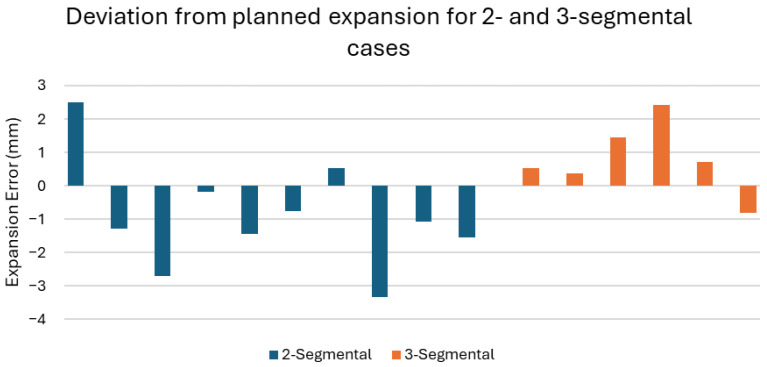
Bar chart of the expansion error for each segmental Le Fort I osteotomy case. The dark blue bars indicate the two-segmental cases, and the orange bars indicate the three-segmental cases. A negative expansion error indicates that the post-operative palatal expansion was smaller than the planned expansion.

Figure 4 illustrates the expansion errors in all 16 segmental cases. In 80% of the two-segmental cases (8 out of 10), the planned palatal expansion was underachieved. Conversely, expansion was overachieved for 83.3% of the three-segmental cases (5 out of 6).

### 3.4. Categorisation of Clinical Accuracy

Optimal outcomes, defined as <1 mm deviation in any direction at the incisor point and <2° deviation in roll/pitch/yaw, were observed in 34.0% (16/47) of the patients (see Table 9). The good accuracy criteria were defined as a translational deviation of 1–2 mm and a rotational deviation of less than 2°, whereupon a total of 17.0% (8/47) of patients met those criteria. A total of 55.3% (26/47) of the patients could be classified as having good osteotomy accuracy, where a rotation accuracy of below 4° was sufficient. The accuracy was classified as suboptimal for 48.9% (23/47) of the cases, where a translational deviation of more than 2 mm in any direction or a deviation of more than 2° in roll, pitch or yaw was present. Translational deviations greater than 2 mm or rotational deviations exceeding 4° were observed in only 10.6% (5/47) of the patients.

## 4. Discussion

### 4.1. General Discussion

This study presents the largest evaluated cohort to date of the accuracy of maxillary repositioning using a two-plate Patient-Specific Osteosynthesis (PSO) system in bimaxillary orthognathic surgery. Our findings demonstrate that this minimally invasive approach achieves submillimetre median translational accuracy. Median rotational deviations for the yaw and roll were below 1 degree. The median pitch deviation presented the greatest challenge (1.6 median inaccuracy) and was the primary cause of suboptimal outcomes. Notably, we found no significant difference in accuracy on comparing one-, two- or three-segment maxillary osteotomies.

Our findings fall well within the established clinical acceptance threshold of 2 mm for orthognathic surgery [1,2,6]. With respect to the noticeable midline deviation (0.4 [0.2–0.7]), the threshold was below 1 mm [3]. This level of accuracy reinforces the clinical viability of two-point fixation at the nasomaxillary buttress, as also found by earlier studies [13,14,15,16]. More recently, Alfaro et al. and van der Wel et al. showed the feasibility of minimally invasive Le Fort I osteotomies using two-plate PSO systems [11,12]. Compared to the largest reported cohort using four-plate PSO systems by Van der Wel et al. [5], our results show that the two-plate system achieves similar accuracy. They reported median deviations of 1.0 mm anteroposteriorly, 0.4 mm mediolaterally, and 0.8 mm superoinferiorly. The median angular differences were 0.5° in yaw, 1.7° in pitch, and 0.5° in roll. Our study achieved equal or better accuracy for all the metrics except roll after using a less invasive approach. Although our study presents a less invasive approach with adequate accuracy, a direct comparison to a control group is not performed and should be incorporated in future work.

Our study identified suboptimal outcomes in 48.9% of the cases (23/47), defined by a translational deviation greater than 2 mm or a rotational deviation exceeding 2°. Using the same criteria, Van der Wel et al. reported a lower rate of suboptimal outcomes (40.5%) with four-point fixation [5]. However, there is no consensus on the threshold for clinically acceptable rotational deviation; while some studies use 2° [1,2,4], others propose a more lenient 4° threshold [6,7]. When applying the 4° criterion, only 10.6% of the cases (5/47) in our study were classified as suboptimal.

### 4.2. Pitch

Despite the overall high accuracy, our results highlight the need for refinement in controlling the pitch deviation. The median pitch deviation was 1.6°, making it the least accurate direction. To explore this observation further, we analysed pitch accuracy separately as counterclockwise (CCW) and clockwise (CW) rotations. In both cases, the planned rotation seemed consistently underachieved, with greater inaccuracy in the CCW direction. One possible explanation is the absence of dorsal fixation plates, which may limit control over the pitch rotation. However, similar challenges were reported by studies of four-point fixation, with pitch deviations of 1.90° by Ho et al., 2.33° by Kraeima et al., 1.85° by Li et al. and 1.7° by Van der Wel et al. [5,17,18,19]. Although pitch was the least accurate of the measured directions in our study, the achieved median deviation of 1.6° still surpasses the pitch accuracy reported by conventional four-point fixation technique studies.

We found a significant correlation between the magnitude of planned movement and the resulting deviation in two directions, pitch and yaw (see Table 7). There was a negative correlation where greater planned movement was associated with reduced accuracy. Given that pitch is already the least accurate rotational axis, particular caution is warranted when planning large pitch adjustments.

### 4.3. Segments and Palatal Expansion

An important contribution of this study is the evaluation of the two-plate system for multi-segmental procedures. The non-significant difference in accuracy across the one-, two- or three-segment cases addresses a gap in the current literature. The accuracy and rigid properties of the two-plate PSO system suggest safe clinical adoption for multi-segmental cases. A limitation of this finding is the small sample size of multi-segmental procedures (*n* = 10 for two-segmental and *n* = 6 for three-segmental cases).

The goal of segmental osteotomies is to achieve transverse palatal expansion or to address the curve of Spee. Our data confirms that the planned palatal expansions (3.0 mm median planned) were largely achieved (2.7 mm median realised), indicating that the two-plate system performs reliably, even in complex cases. We observed a median absolute error of 1.2 mm. This represents a substantial proportion of the targeted expansion. Further research is necessary to establish whether a clinical threshold exists to guide the decision for or against performing a two-plate segmental osteotomy for widening purposes.

The observed deviations in palatal expansion between the two- and three-segmental cases were noteworthy. In 80% of the two-segmental cases (8/10), the planned expansion was underachieved, whereas it was overachieved in 83.3% of the three-segmental cases (5/6). This trend may suggest that two-segmental osteotomies tend to result in conservative expansion outcomes, while three-segmental procedures may carry a tendency towards overexpansion. Further research with a larger cohort is needed to determine whether this trend is reproducible.

### 4.4. Longer-Term Stability of the Result

Post-operative evaluation was conducted by our study 7–10 days after the surgery. This does not address the long-term skeletal stability of our results. A recent mixed-methods review by Grillo et al. compared two- and four-plate fixation in Le Fort I osteotomy [20]. A meta-analysis of clinical trials showed no significant difference in stability between the two fixation methods. However, a subsequent biomechanical analysis (in silico and in vitro) revealed an unusual increase in the risk of relapse following two-plate fixation, starting with 3 mm maxillary advancements. Grillo et al. suggested this might be due to the four-plate configuration’s ability to dissipate force over a broader area. Importantly, several of the studies used by Grillo to support this conclusion compared conventional L-plates with two or four-plate fixation in their biomechanical data, rather than the adjusted larger Y-plates used in our study and others. The increased fixation points in our plates, compared to the standard L-plate, combined with a larger bone contact surface area, may help the inverted Y-plate maintain stability. Nonetheless, further research is necessary to investigate the long-term postoperative skeletal stability of the current two-plate system.

### 4.5. Exclusion of CBCT Scans Due to Stitching Error

A limitation of this study is the exclusion of 14 patients from the final analysis due to movement artefacts observed in their CBCT scans. The exclusion of a substantial proportion of the initial cohort raises concerns regarding selection bias and the potential impact on the generalizability of our findings. However, the mechanism for exclusion was purely technical, related to an imaging protocol used for some of the included cohort, and is unlikely to bias the results. Initially, all the patients were scanned using a separate lower- and upper-face scan, after which the scans were automatically stitched together in the scanners’ software. During the analysis, we noticed artefacts in the stitching regions. After discovering the error, the imaging protocol was changed to a single large VOF CBCT protocol without stitching.

## 5. Conclusions

This study confirms that a two-plate Patient-Specific Osteosynthesis (PSO) system enables maxillary repositioning with submillimetre accuracy following bimaxillary surgery. Its reduced invasiveness and hardware requirements make the presented system a promising alternative for treating dentofacial deformities. Accuracy was maintained across one-, two-, and three-segment maxillary osteotomies, suggesting that clinicians may safely adopt two-plate fixation for both standard and complex multi-segmental cases. However, further research is needed to evaluate their long-term stability and effectiveness in pitch correction.

## Figures and Tables

**Table 1:** Patient inclusion and exclusion criteria.

Inclusion Criteria	Exclusion Criteria
Patient underwent a bimaxillary osteotomy	Unsuitable CBCT quality for analysis.
Patient was operated with a two-plate PSO system	
Availability of both pre- and post-operative CBCT scans.	

**Table 2:** Criteria for categorising individual outcomes into optimal, good, or suboptimal based on translational and rotational deviations from the Virtual Surgical Plan, utilising two different rotational thresholds (2° and 4°) for clinical acceptability.

Result	2° Criteria	4° Criteria
Optimal	<1 mm deviation in translation AND <2° deviation in roll/pitch/yaw	<1 mm deviation in translation AND <2° deviation in roll/pitch/yaw
Good	1–2 mm deviation in translation AND <2° deviation in roll/pitch/yaw	1–2 mm deviation in translation AND <4° deviation in roll/pitch/yaw
Suboptimal	>2 mm deviation in translation OR >2° deviation in roll/pitch/yaw	>2 mm deviation in translation OR >4° deviation in roll/pitch/yaw

**Table 3:** Demographics of the 47 patients included in the final analysis, categorised by the number (No.) of segments in the maxillary osteotomy.

Demographic	
No. of patients	47
Mean age (years ± SD)	27.9 ± 9.4
No. of one-segment maxillary osteotomy patients	31
No. of two-segment maxillary osteotomy patients	10
No. of three-segment maxillary osteotomy patients	6

Abbreviation: SD, standard deviation.

**Table 4:** The median planned movement of the maxilla for the 47 included patients. Translation movement is reported in mm and rotational movement in degrees. Directions of movement are indicated for each parameter.

Median Planned Movement of the Maxilla	
Direction	Median [IQR]
Ant/Post (mm)	5.0 (Ant) [3.8 (Ant)–6.5 (Ant)]
Left/Right (mm)	0.0 [1.0 (Left)–0.0 (Right)]
Up/down (mm)	0.0 [0.0 (Up)–2.0 (Down)]
Roll (°)	0.1 (CCW) [1.8 (CCW)–1.5 (CW)]
Pitch (°)	0.9 (CW) [1.7 (CCW)–4.9 (CW)]
Yaw (°)	0.3 (CCW) [0.7 (CCW)–1.8 (CW)]

Abbreviations: Ant, anterior; Post, posterior; CCW, counterclockwise; CW, clockwise; IQR, interquartile range.

**Table 5:** Absolute deviations from the planned position of the maxilla after bimaxillary surgery using a two-plate PSO system.

Direction	Median [IQR]
Ant/Post (mm)	0.7 [0.4–1.2]
Left/Right (mm)	0.4 [0.2–0.7]
Up/down (mm)	0.6 [0.2–1.1]
Roll (°)	0.8 [0.5–1.2]
Pitch (°)	1.6 [0.6–2.4]
Yaw (°)	0.5 [0.3–1.2]

Abbreviations: Ant, anterior; Post, posterior; IQR, interquartile range.

**Table 6:** Spearman’s correlation between the magnitude of the planned movement and the realised deviation from the planning.

Direction	Correlation (*r*-Value)	*p*-Value
Ant/Post (mm)	0.080	0.592
Left/Right (mm)	0.285	0.052
Up/down (mm)	−0.127	0.395
Roll (°)	0.311	0.034
Pitch (°)	0.316	0.031
Yaw (°)	0.201	0.175

Abbreviations: Ant, anterior; Post, posterior.

**Table 7:** Absolute deviations from the planned position of the maxilla across three landmarks: upper incisor, upper canine and upper first molar. The deviations are displayed for each number of segments used in the Le Fort I osteotomy.

Landmark	Direction	1-Segment (*n* = 31)	2-Segment (*n* = 10)	3-Segment (*n* = 6)	*p*-Value
		Median [IQR]	Median [IQR]	Median [IQR]	
Upper Incisor	Ant/Post (mm)	0.7 [0.4–1.3]	0.9 [0.5–1.4]	0.5 [0.3–0.7]	0.279
	Left/Right (mm)	0.5 [0.2–0.7]	0.5 [0.2–1.2]	0.3 [0.1–0.8]	0.596
	Up/Down (mm)	0.6 [0.3–1.1]	0.9 [0.5–1.3]	0.4 [0.1–0.9]	0.209
Upper Canine	Ant/Post (mm)	0.7 [0.4–1.3]	1.3 [0.5–1.6]	1.1 [0.6–1.9]	0.420
	Left/Right (mm)	0.4 [0.1–0.7]	0.4 [0.3–1.1]	1.0 [0.5–1.6]	0.060
	Up/Down (mm)	0.5 [0.2–0.7]	0.7 [0.4–1.1]	0.6 [0.4–1.5]	0.873
Upper First Molar	Up/Down (mm)	0.8 [0.4–1.3]	1.2 [0.5–1.8]	0.9 [0.6–1.5]	0.263
	Left/Right (mm)	0.4 [0.2–0.7]	0.7 [0.5–1.6]	0.5 [0.3–1.3]	0.065
	Up/Down (mm)	0.8 [0.5–1.4]	0.8 [0.3–1.4]	1.4 [0.3–1.5]	0.634

Abbreviations: Ant, anterior; Post, posterior; IQR, interquartile range.

**Table 8:** Analyses of the planned and released expansion for the 16 segmental osteotomy cases, measured as the distance between the first upper molars.

Planned Expansion (mm)	Realised Expansion (mm)	Absolute Error (mm)
Median [IQR]	Median [IQR]	Median [IQR]
3.0 [1.8–3.8]	2.7 [1.3–3.2]	1.2 [0.6–2.2]

Abbreviation: IQR, interquartile range.

**Table 9:** Summary of all patient accuracy results, categorised as optimal, good or suboptimal according to the criteria shown in Table 2. The results are presented according to two different rotational criteria for suboptimal outcomes: 2° and 4° deviations.

Result	2° Criteria	4° Criteria
Optimal	34.0% (16/47)	34.0% (16/47)
Good	17.0% (8/47)	55.3% (26/47)
Suboptimal	48.9% (23/47)	10.6% (5/47)

## Data Availability

The Data presented in this study are available on request from the corresponding author due to privacy/ethical restrictions.
